# Passive buildings: a state-of-the-art review

**DOI:** 10.1186/s43065-022-00068-z

**Published:** 2023-01-11

**Authors:** Vishwajit Anand, Vishnu Lakshmi Kadiri, Chandrasekhar Putcha

**Affiliations:** 1 Department of Civil Engineering, Indian Institute of Technology (Banaras Hindu University), Varanasi, 221 005 India; 2 Department of Civil Engineering, National Institute of Technology Andhra Pradesh, Tadepalligudem, 534 101 India; 3 Department of Civil and Environmental Engineering, California State University, Fullerton, CA 92834 USA

**Keywords:** Passive buildings, Energy conservation, Emission reduction, Economic feasibility, Lifecycle cost assessment, Climatic adaptability

## Abstract

Passive buildings are proving to be a solution to menaces of energy crisis and greenhouse gas emissions across the world. Such buildings tend to exhibit low energy demand owing to their cleverly designed envelopes, which comprise of walls, roofs, doors, windows and other openings. This requires use of new materials and technology, leading to an increased initial construction cost. However, with reduced energy consumption, the lifecycle cost of a passive building may be lower than that of a conventional building. These passive buildings also need to cater to occupants’ comfort which is subject to local climatic conditions and climate change. This article discusses economic feasibility and climatic adaptability of a passive building, in addition to advances in passive building strategies. Owing to lack of general awareness and standards related to passive building construction, these buildings have not achieved enough popularity. While many countries are striving hard to bring passive buildings to common masses, a large number of countries are yet to initiate the move. This article outlines several active organizations, standards and rating systems for passive buildings. This article also presents some of the recent research trends and a comprehensive bibliography for the benefit of researchers and practitioners.

## Introduction

**Fig. 1:**
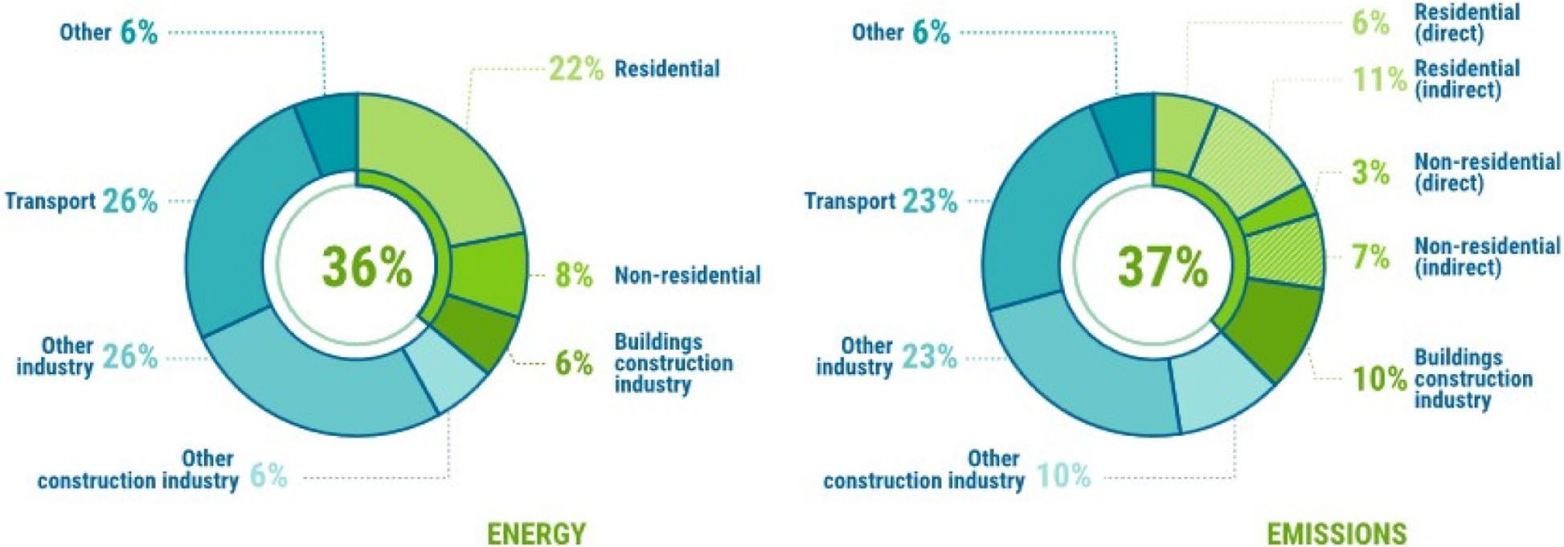
Contribution of buildings and building construction industries in global energy consumption and greenhouse gas emissions. Source: UNEP [4]

Unsustainable development in the post-industrial revolution era has resulted in an ever-growing energy demand and greenhouse gas emissions, across all sectors including buildings, transport and industries. This has led to change in climate across the globe, with noticeable extreme weather phenomena and rising mean sea levels. The rate of climate change has been observed to increase steadily over last four decades [1, 2]. As a result, 196 parties comprising mostly of nations entered into the Paris Agreement of 2015, which is a legally binding international treaty on climate change. It was agreed that the global mean temperature rise be restricted to 2 °C over a century [3]. It has also been evident that with gradual shift in nature of occupations from farms to indoors, buildings have turned into a major contributor towards energy use and greenhouse gas emissions. Globally as of 2021, buildings and building construction industry account for 36% and 37% of energy and emissions respectively as shown in Fig. 1 [4]. The contribution of a paradigm shift in nature of jobs can be understood if these numbers for countries at different levels of the development ladder are assessed. For instance, in 2019, buildings’ share in total energy consumption in countries in Asia barring China and those in Central and South America was in the range of 23–25%, which is lesser than the global levels by almost 12 percentage points [4]. However, there are multiple factors such as energy source for domestic usage, efficiency of transport network and levels of industrialization, which influence buildings’ share in total energy consumption. On an average, buildings and building construction industry in African countries together contributes to 60% of total energy consumption. The region’s high dependence on bioenergy for its domestic energy needs is attributed to this high share of buildings in total energy consumption [4]. Another important perspective is the rapid growth in buildings’ share in total energy consumption for the developing countries such as India and Brazil as compared to United States and European nations, for the period 1990–2010 [5]. It can be assessed that the developing nations should learn from the examples of developed nations and thereby act pro-actively to avoid getting into deeper waters. The effect of population growth and urbanization on buildings’ share in energy consumption and greenhouse gas emissions is also worth noting [5]. Despite the variation in these shares across nations, the global average share of 36% and 37% to energy consumption and emissions makes it crucial to design and construct energy efficient and environment friendly buildings in order to achieve goals set by the Paris Agreement. The annual reports indicate that the present development in this direction has not been satisfactory [4, 6], as only 80 countries have either mandatory or voluntary building energy codes at national and/or sub-national levels [4]. In order to improve the acceptability of the need to switch to energy efficient and environment friendly buildings, awareness related to such buildings among masses is required. People should be made aware of vernacular and modern strategies to improve energy efficiency and reduce carbon footprint in buildings, which will enable them to make informed choices. These strategies can be either active or passive [7, 8]. Though this article reviews elements of passive buildings in particular, understanding of these two types of strategies is important.

Most of the building projects in the developing world still award the contract to the option with lowest initial cost. The significance of considering lifecycle cost as the criterion is now being understood, but unavailability of reliable data, divergence in lifecycle costs standards, and lack of awareness among vast majority of population hinder the use of lifecycle cost as a deciding parameter in most projects [9]. It is intuitive that initial costs of energy efficient and environment friendly buildings is usually higher than conventional buildings. However, with reduced energy consumption and shrunk carbon footprint, such buildings are likely to have a lower cost over its design life. However, in a bid to make a building highly energy efficient and environment friendly, a design may be arrived which may not render the building economical in its design life. Such designs are examples of over-investment and should be avoided. It is therefore imperative to arrive at the optimal level of investment in energy efficiency and emission reduction [10], which would imply one to understand how to estimate lifecycle cost for buildings.

It is also worth noting that both active and passive strategies for improving energy efficiency and reducing emissions in case of buildings are popular in North America and Europe, particularly among Scandinavian countries. Though it may be attributed to high per capita income in these countries making initial expense affordable to a majority of population, the climate also seems to have a role. For instance, buildings in cold climates have to employ strategies which would reduce energy consumed in heating the indoor space. However, in a sub-tropical and/or temperate climatic zone, buildings need to employ energy efficiency strategies for heating in winters and cooling in summers. This would mean additional investment which may lead to lifecycle cost for the energy efficient building higher than that for the conventional structure. This implies the necessity of studying climatic suitability of the proposed investment [11, 12]. Vernacular construction practices in the region are likely to provide a good solution, and they need to be explored as well.

Based on above preamble, this article has the following objectives: (i) to review different strategies to improve energy efficiency and reduce emissions in buildings, with emphasis on passive buildings (Energy efficient and environment friendly buildings), (ii) to outline procedure for lifecycle cost assessment for passive buildings (Economic feasibility of passive buildings), (iii) to understand adaptability of passive buildings to diverse climatic zones and climate change (Climatic adaptability of passive buildings), (iii) to outline prominent standards related to construction of passive buildings (Standards on passive buildings) and (iv) to discuss present shortcomings and thereby explore novel research opportunities (Contemporary research). The article is likely to provide readers a holistic insight into the world of passive buildings, and thereby enable them to make informed choices while planning to build energy efficient and environment friendly buildings. The article will also help researchers in developing novel strategies for further improving energy efficiency and reduce greenhouse emissions, in case of buildings. The article hosts a good bibliography which will enable readers develop knowledge and understanding specific to their research interests.

## Energy efficient and environment friendly buildings

Energy efficient and environment friendly buildings are commonly termed as green or sustainable buildings. United States Green Building Council (USGBC) defines green buildings as holistic buildings, which in planning, design, and operation have a positive effect on their surroundings. Such buildings consume minimum natural resources for their construction and operation throughout their design life, promotes reuse, recycling and utilization of renewable resources, and thereby reduce our dependence on non-renewable resources [13].

There are diverse strategies to reduce energy consumption and greenhouse gas emissions in buildings, and to achieve green building standards set by different agencies such as USGBC. Technological improvements to heating, ventilation and air conditioning (HVAC), lighting, electrical and plumbing systems are classified as active strategies. On the other hand, passive strategies include fundamental changes in the building envelope [8, 10]. Building envelope comprises of all the elements that separate the indoor environment from the transient outdoor environment. Some of the common tangible elements of building envelope are walls, roofs, foundations, doors, windows, sunshades and external façade. Though thermal mass and thermal insulation of a building are intangible, they contribute towards making the building energy efficient and environment friendly. Therefore, these properties are also considered as components of building envelope. The last decade has witnessed an ever-increasing interest in employing passive strategies to build energy efficient and environment friendly buildings, and these strategies are being envisioned as solutions to the menaces of energy crisis and environmental pollution [8]. A number of recent studies from across the globe have observed a lowering in energy consumption by 30 to 50% because of changes in the building envelope such as thermal insulation in roofs and walls, light shaded external walls and roofs, overhang and wing walls in windows, and reflective coated glass window glazing [14–16]. This lowering can however vary greatly depending on building envelope choices and location [17, 18]. These passive strategies make the indoor environment comfortable without a need to over-rely on HVAC systems, resulting into lower greenhouse gas emissions. Architectural innovations like adequate orientation of a building and use of self-shading elements are also very effective passive strategies which improve lighting and ventilation of the building, and thereby reduces the energy consumption [19–22]. Natural daylighting and ventilation also have positive effects on health and mood of occupants [23–26]. This would lead to happier families in residential buildings and improved productivity in office and commercial buildings [23–26]. The efficacy of a building envelope is usually assessed using a numerical study of heat transfer through the envelope by various modes, viz. conduction, convection and radiation [27–29]. Solar collection envelope of a building provides further information about variation in solar gain throughout the year, and can therefore be beneficial in blocking the summer sun while permitting the winter sun [19]. The following sections outline typical passive elements that can be included in prominent building envelope components such as walls, roofs, doors, windows, sunshades and façade.

Passive buildings are frequently confused with Net Zero Energy (NZE) buildings. NZE buildings are able to produce energy that it consumes, through renewable sources (usually solar), on an annual basis. Typically, these buildings produce extra energy during summers, sell it to the grid, and purchase the same during winters when the solar installation is unable to meet the demand. Some of these buildings can be net positive energy buildings where the energy generation exceeds its demand over a year. This means NZE buildings are environment friendly as they lead to lower emissions in electricity generation. However, unlike passive buildings, reduction in energy consumption in buildings is rarely the aim of NZE buildings. On the other hand, passive buildings aim to lower their energy consumption by exploring changes in their building envelopes. This is the reason why passive buildings are presently considered a real solution to energy crisis and environmental pollution. Furthermore, passive buildings also tend to improve comfort and elevate mood of the occupants.

### Walls

Walls and roofs are the most prominent tangible components of a building envelope. The aspect ratio of the buildings determines their relative significance in case of passive buildings. While a typical squat building has relatable significance of walls and roofs, walls of high-rise buildings are much more significant than its roof. Passive building design involves walls and roofs that insulate the indoor and outdoor environments to make the indoors habitable and comfortable. At times, walls also serve the purpose of illumination and letting daylight enter the building.

**Fig. 2:**
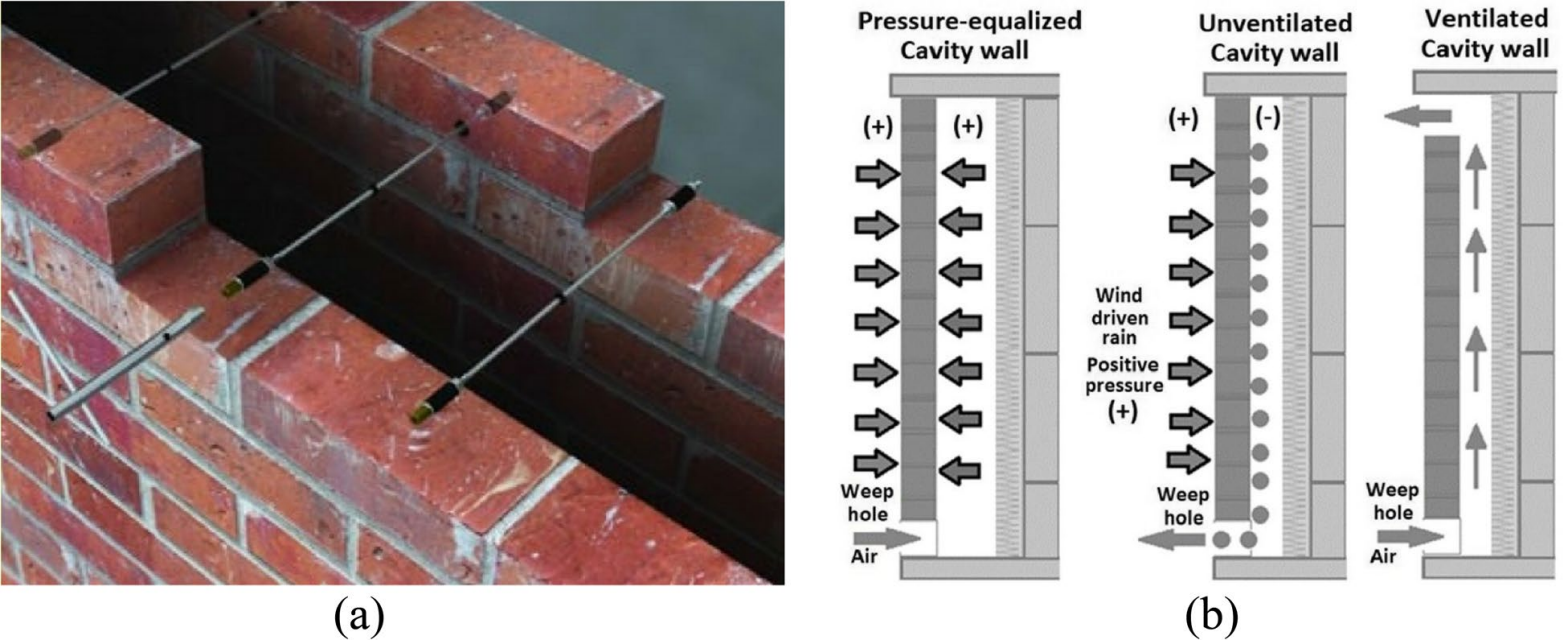
**a** A typical cavity wall, **b** Different types of cavity walls. Source: Williams [36]

Traditional construction in Indian subcontinent involved walls made of stones, mud and/or adobe, which have poor thermal conductivity and absorption capacity [30–33]. However, with urbanization and need for higher buildings, this has been replaced with concrete and/or masonry walls. Concrete walls tend to absorb a lot of heat and emit it very slowly, leading to overheated indoor spaces in tropical regions during summers [34]. Another classical solution is to use cavity walls shown in Fig. 2. Such walls comprise of two masonry wythes/skins (external and internal) separated by an air cavity, and are therefore also called double skin or ventilated walls [35–37]. It has been observed that increase in width of air cavity improves insulation and thereby energy performance. However, this benefit gets saturated at a cavity width of 150 mm [38]. The relative roughness and thermal resistance of the two layers also influence the performance of cavity walls, and the performance is usually characterized by *R*-value [39].

Modern buildings are primarily framed structures with columns as their vertical load bearing element. These buildings can use lightweight concrete for walls. Lightweight concrete used for thermal insulation in walls have mass density around 1400 kg/m^3^ and have significantly lower strength compared to concrete used for regular construction. Lightweight concrete is either made by use of lightweight aggregates and/or entrainment of air. Suitable aggregates for this purpose can be obtained from natural materials or industrial by-products. Air entrainment is obtained by using a chemical admixture or introducing aluminium powder. When aluminium powder is used to obtain lightweight concrete, it is referred as autoclaved aerated concrete (AAC). AAC blocks can be easily manufactured in a factory and are therefore suitable for precast construction. This makes them a good choice for use in rapid construction of passive buildings. A recent innovation is use of phase change material (PCM) in the lightweight concrete or gypsum walls [40–42]. Such walls are termed as walls with latent heat storage and their efficacy depends on the amount of impregnated PCM material. Research shows that walls with PCM result in considerable isolation of indoor environment from the outdoor, and thereby reduce the demand on HVAC systems [41, 43, 44].

**Fig. 3:**
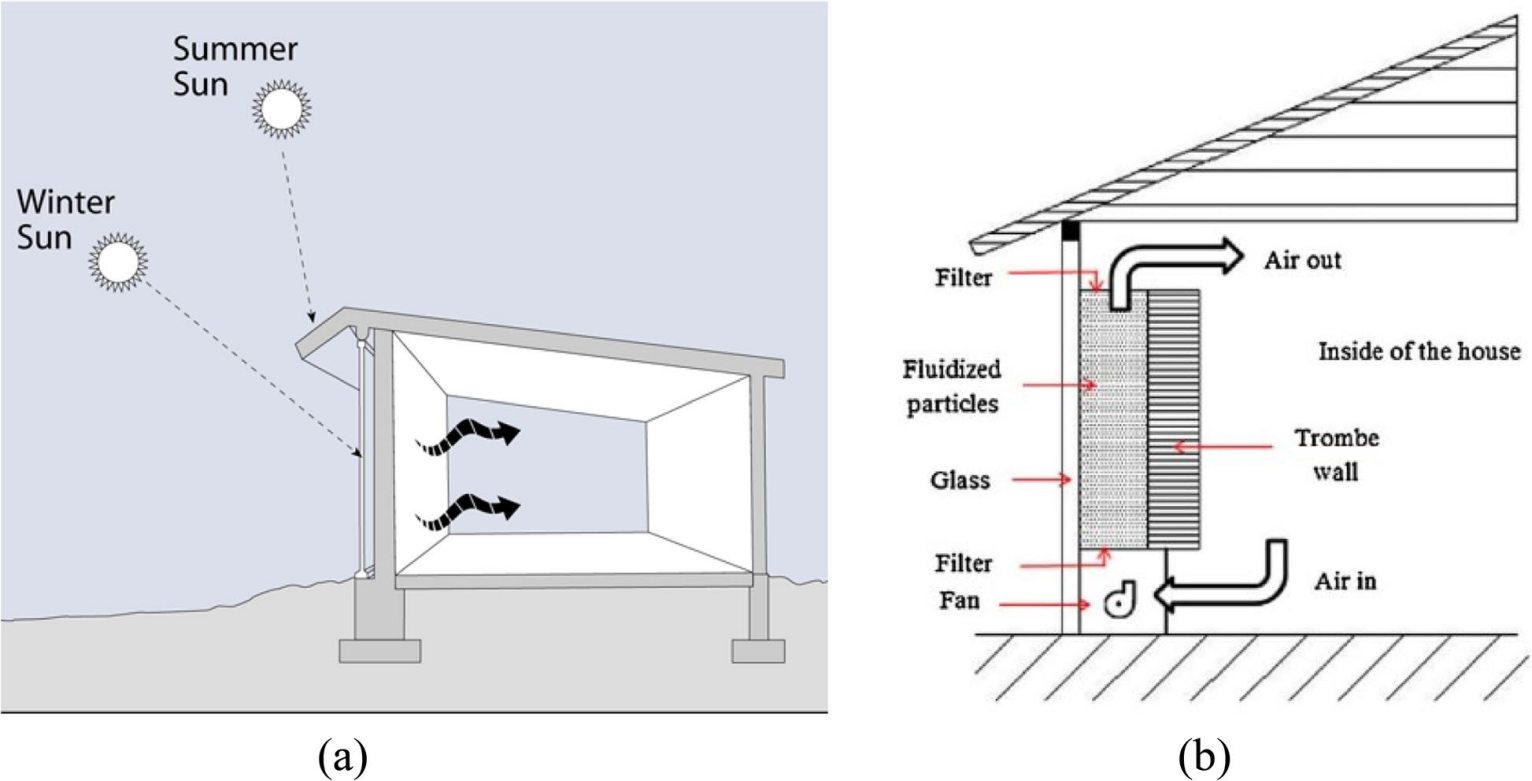
**a** A typical passive solar wall (Trombe wall), **b** Fluidized Trombe wall. Source: Tunc and Uysal [56]

However, the most commonly used wall technology in passive buildings is a passive solar wall. A classic passive solar wall, commonly called unventilated solar wall, consists of a 12-in. concrete wall on the southern and northern face of buildings in northern and southern hemispheres respectively. This orientation enables the building to absorb solar radiation in winters during the day, and release the same at night to make the indoor environment habitable. Researchers led by Trombe redesigned these walls by adding a glazing on the outer side of the wall to provide the greenhouse effect, and this wall system is termed as Trombe wall and is shown in Fig. 3 [45]. A number of improvements from the basic design of Trombe walls to improve efficacy of the building envelope, can be observed over the decades [46–49]. Since these walls are designed to absorb solar radiation and thereby suit cold climatic zones, they may lead to overheating in tropical zones. To avoid overheating, these walls may be adequately insulated resulting into isolated Trombe walls. The insulation may be a permanent fixture or an operable solar shield [50, 51]. An innovative feature is to affix photovoltaic (PV) cell array between concrete wall and glazing [52, 53]. As a result, Trombe wall absorbs solar radiation which is not consumed by the PV cell array. The energy stored in the PV cell array can be used for indoor lighting purposes leading to reduction in energy demand from the grids. PCM based Trombe walls have been observed to be thinner than the conventional Trombe wall [54, 55]. A recent innovation in the class of Trombe walls is fluidized Trombe wall [56, 57]. This system comprises of a fluid with highly absorbing, low-density particles filling the gap between concrete wall and glazing of the Trombe wall system. The solar energy is released into the building through a filter, which is then circulated through fans. Since this system makes use of conduction, convection and radiation unlike conduction alone in the case of classic Trombe wall, fluidized Trombe walls are highly efficient. There have been a lot of recent innovations in Trombe walls depending on the project objectives [57, 58].

If the building requires both heating and illumination, a transwall, comprising of two parallel glass panes with water enclosed between them, is very efficient [59, 60]. A translucent glass pane is often placed at the centre of parallel glass panes. While a part of the incident solar radiation is absorbed by water and translucent glass pane, the rest causes heating and illumination of the indoor environment [59].

### Roofs

For large roof structures such as auditoria, exhibition halls and indoor stadia, roof can be designed as an adequate thermal exchange or barrier. This makes roof of such buildings to be the most significant component of the building envelope.

The traditional roof construction in Indian subcontinent used burnt clay units with mud mortar covered with a layer of terracotta tiles [61]. This design has been very successful at shielding the indoor environment from the outdoor environment which can be extreme in tropical regions. However, in a bid to go higher, most modern buildings are using concrete slabs which have high solar absorption and longer heat retaining capacity. This tends to overheat the dwelling spaces in summers, and in turn increase energy consumption by HVAC systems [61, 62]. As a consequence, a number of modifications such as roof shadings, roof coatings or compound roof systems, have been attempted [63–65]. Another common roof in case of buildings with large plan area is lightweight aluminium standing seam roof. These roofs also have poor thermal characteristics, and need to be adequately insulated. The insulation layer can be composed of glass fiber, polyurethane, polystyrene or a mix of these, depending on the climatic zone of application [62, 66].

**Fig. 4:**
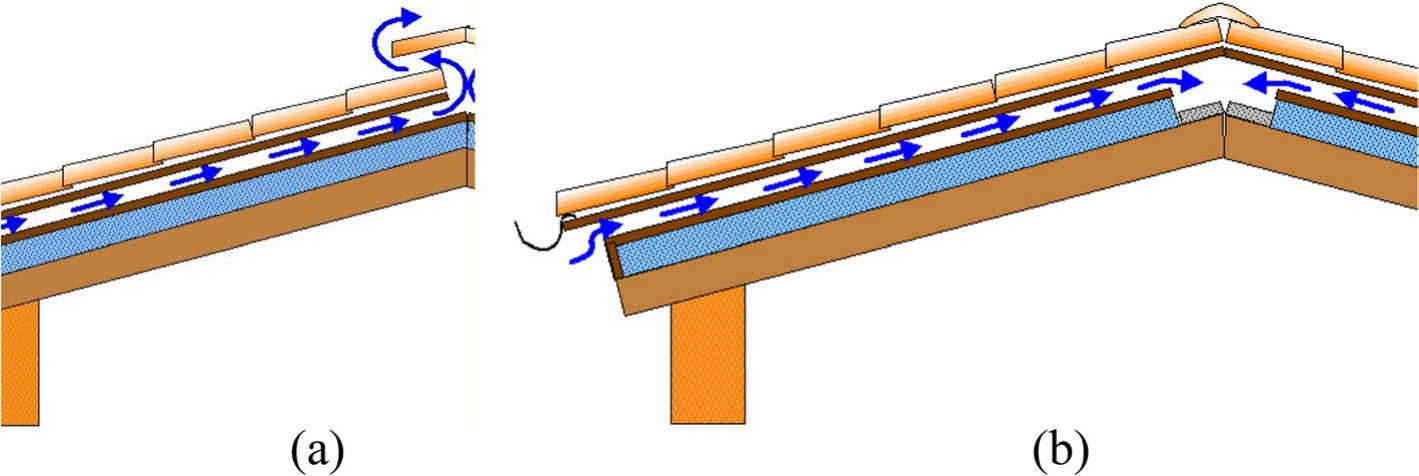
**a** Pitched ventilated roof with natural ventilation, **b** Pitched ventilated roof with forced ventilation. Source: Ferrari and Muscio [69]

Similar to cavity walls, a roof can also be composed of two slabs with an air cavity between them. Such roofs are called ventilated roofs. The air flow between the roof slabs limits the heat transfer across the roof, leading to a habitable dwelling space in summers. The air cavity is usually sealed during winters to allow solar heating. These ventilated roofs are popular in hot and humid climate, and have the potential to lower energy demand by 30% [67, 68]. These roofs can provide natural or forced ventilation as shown in Fig. 4, depending on local climate [69].

In hot and arid climate, vaulted and domed roofs are effective in quickly dissipating the absorbed heat when the outdoor temperature falls sharply at night [70–72]. Furthermore, most of the thermal gradient occurs within the vault or dome volume, rendering the dwelling space cooler. Provision of cross-ventilations near the base of vault is also helpful in getting rid of the hot air. The vaults are usually oriented north-south for an improved performance, with the rim angle usually exceeding 100°.

Thermal characteristics of roofs can also be improved using roof coatings which have high solar reflectance and emittance properties. The coating can be either white paint, PVC membrane, elastomer or aluminium. The efficacy of these roofs increases with coating thickness [73–75].

There is a recent trend to have green roofs in a building, which essentially means having a cover of vegetation to avoid excessive solar heating [76]. A green roof comprises of conventional roof, waterproofing membrane, drainage, root barrier layer, soil and vegetation, from bottom to top [77]. The existing roof can also be easily transformed into a green roof as the total load of all layers is well within the design load [78]. The moist soil medium improves insulating behaviour of the green roof by acting as a thermal barrier [79–81] as well as promoting evaporative cooling [82]. These roofs are popular in commercial and office buildings where the roof may not be accessible except for maintaining the vegetation growth. Green roofs are suitable in diverse climatic zones [83–86]. Evaporative roof cooling can also be achieved by astutely ponding the roof or using hessian/jute bags soaked in water [62, 63, 87].

Recent times have witnessed significant strides in constructing photovoltaic (PV) roofs. These roofs either comprise of PV roof tiles or modules [88, 89], depending on accessibility of the roof to the occupants and choice of roofing system. While acting as thermal shields, PV roofs also produce electricity from renewable solar energy, leading to reduced dependence on grids. PV roofs can be integrated with green roofs for an enhanced energy saving [90, 91].

Roofs in buildings with large plan area also serve as source of illumination and daylighting. Vaulted roofs, with daylighting from south in the northern hemisphere and vice-versa, are usually employed for this purpose.

### Openings (doors and windows)

Doors and windows play a vital role in buildings for access, thermal comfort and optimal illumination of the indoor environment. Properly laid doors and windows also add value to the aesthetics of the building. Recent decades have witnessed advent of numerous glazing technologies such as solar control glass panes, insulating glass panes, low emissivity and reflective coatings, vacuum glazing and gas filled glazing systems. Their applications depend on their thermal conductivity and solar heat gain capacity, in addition to their orientation, building characteristics and climate of the region [92–94]. Thermal insulation of openings is characterized by *U*-value, which is mathematically reciprocal of *R*-value for walls and roofs. While higher *R*-value indicates better thermal resistance, lower *U*-value implies better thermal insulation.

Aerogel glazing reduce heat gain by transforming the window pane [95–97], and would be suitable in summers. However, buildings in tropical regions may need heat gain in winters without a need to open the windows. This is achieved by use of switchable reflective glazing, which uses a low DC voltage or a gas like hydrogen to change from transparent to tinted state and thereby restrict the solar gain [95, 98–100].

Similar to cavity walls and ventilated roofs, some passive buildings use two glass panes with vacuum between them, in order to eliminate heat transfer across the window by conduction and convection [98, 101]. The vacuum can also be filled with inert gases like argon. A recent innovation is use of light absorbing suspended particles in the cavity between the glass panes [95, 102]. These suspended particles align in a defined orientation to prevent heat and light exchange. This transition occurs quickly with a trigger coming from a switch.

**Fig. 5:**
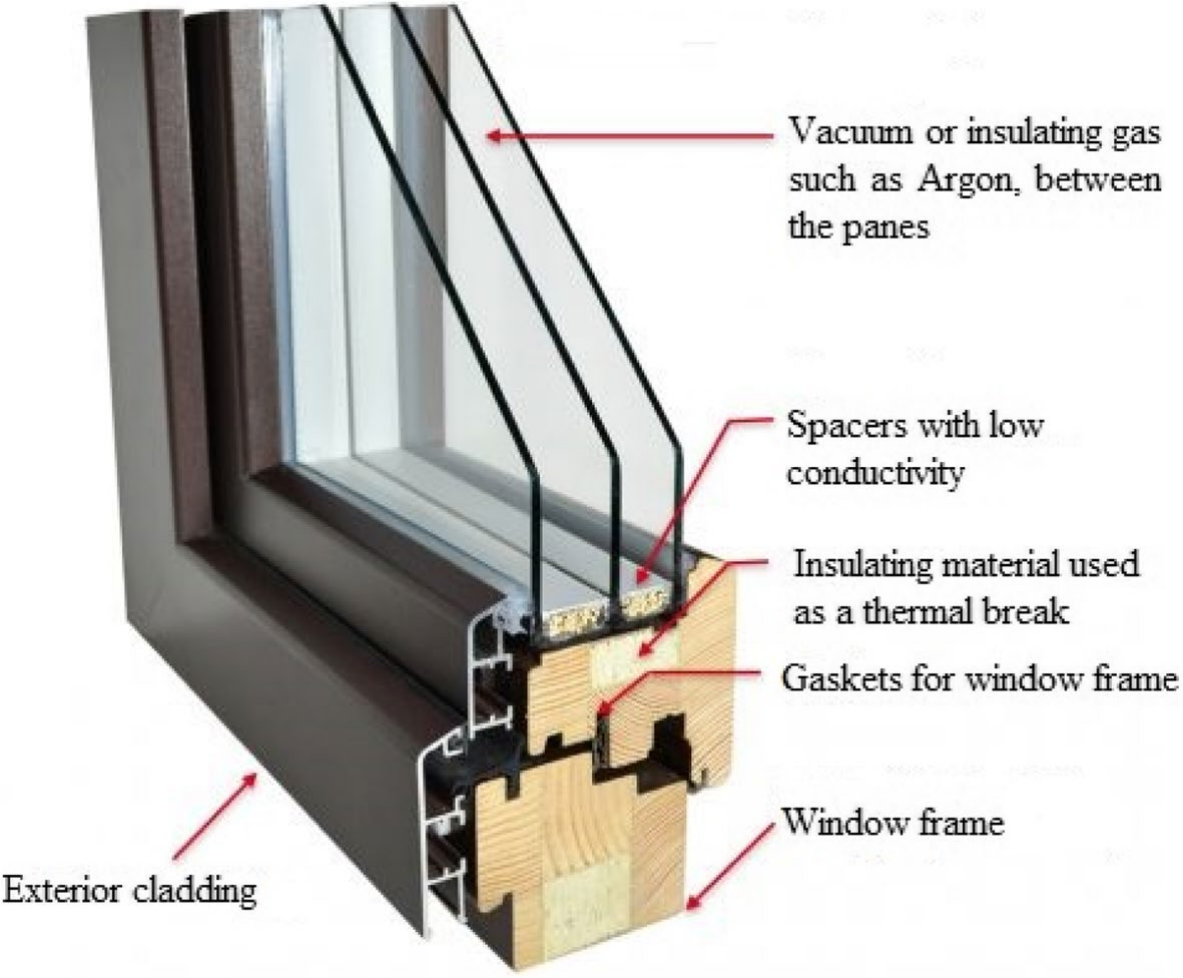
A typical multi-pane window for insulating indoor spaces

It is intuitive that the insulating behaviour of an opening is as good as its supporting frame. The edges of doors and windows must be adequately sealed as shown in Fig. 5, to get the desired performance from the glazing. Edge effect are more dominant in case of smaller size openings [92].

From the above discussion, it is evident that the choice of technology for walls, roofs and openings depend on the desired effect which in turn heavily depends on the climate of the site. It is obvious that use of any of these technologies is likely to increase cost of construction of the project. However, the initial investment will result into an energy efficient and environment friendly building, which will certainly reduce the cost of operating and maintaining the building. In the upcoming section, we will discuss how to estimate lifecycle cost of a building. Estimation of lifecycle cost would enable us to decide the level of optimal investment in passive buildings.

## Economic feasibility of passive buildings

It is well established that passive buildings are solutions to menaces of energy crisis and ecological damage posed by the built environment. However, since construction is capital intensive, it is utmost important that solutions be evaluated for economic feasibility. This is much more relevant in case of residential buildings in developing world where people tend to spend much more than their liquid assets and end up taking institutional and non-institutional loans. These construction loan portfolios attract very high loss rates during economic downtime and are a key factor in failure of many banks [103]. The severe economic threat posed by recent pandemic COVID-19 has forced individuals as well as federal governments to think in this direction.

As a consequence of loans’ principal amount and its associated interests, most buildings till date are being built up considering only the initial cost of construction. Since passive buildings use modern technologies in their envelopes, their initial costs are usually higher than conventional buildings [104]. Among multiple barriers, this is one of the primary reasons behind poor acceptance of passive buildings among various stakeholders of building construction including clients, governments, design and construction engineers, and financing institutions [104, 105]. However, owing to lower energy consumption, passive buildings tend to have reduced operational costs and are therefore a viable investment [104]. Furthermore, government institutions have been granting subsidies for private passive constructions as they tend to reduce carbon footprint of the region [106, 107]. It is therefore intuitive to conclude that passive buildings may end up being economical than conventional buildings in long run. This implies the significance of assessing economic feasibility of proposed passive construction before it is deemed fit to be built.

### Lifecycle cost estimation of passive buildings

Economic feasibility is usually assessed by estimation of lifecycle costs (LCC) associated with the building, which is the total cost of owning, operating, maintaining, and disposing of the building over a given study period. In order to compare economic feasibility of multiple passive alternatives against conventional non-passive construction (usually called base case), the future costs of operation, maintenance and disposal are converted to their present value equivalents. This process is called discounting, and is achieved using Eq. (1), where PV and FV respectively denote present value and future value after *t* years, and *d* denotes the discount rate. The process of obtaining compound interest forms the basis of the discounting process.

1 $$PV=\frac{FV}{{\left(1+d\right)}^t}$$

Discount rate refers to investor’s minimum acceptable rate of return, and should not be confused with rate of inflation. Since early investment means a loss of opportunity of investing the capital somewhere else, the investor is more interested in expected rate of return had he/she invested in open markets. Since expected rate of return is used, LCC for various design alternatives is said to be estimated rather than computed. Though discount rate is a function of risk and benefit appetite of the investor, some agencies in the United States recommend lower and upper rates of discount rates [108–110].

**Fig. 6:**
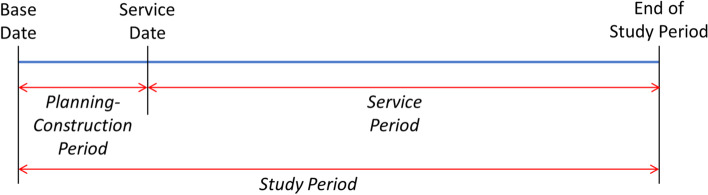
Description of study period used in economic feasibility analysis of passive buildings

The study period for LCC refers to time over which costs and benefits related to a capital investment decision are of interest to the investor. Depending on investment routine and habit of the investor, the study period may be significantly less than the design life of the building. However, it must be considered the same for all the design alternatives being considered. The study period can be divided into planning-construction period and service period, as depicted in Fig. 6.

**Fig. 7:**
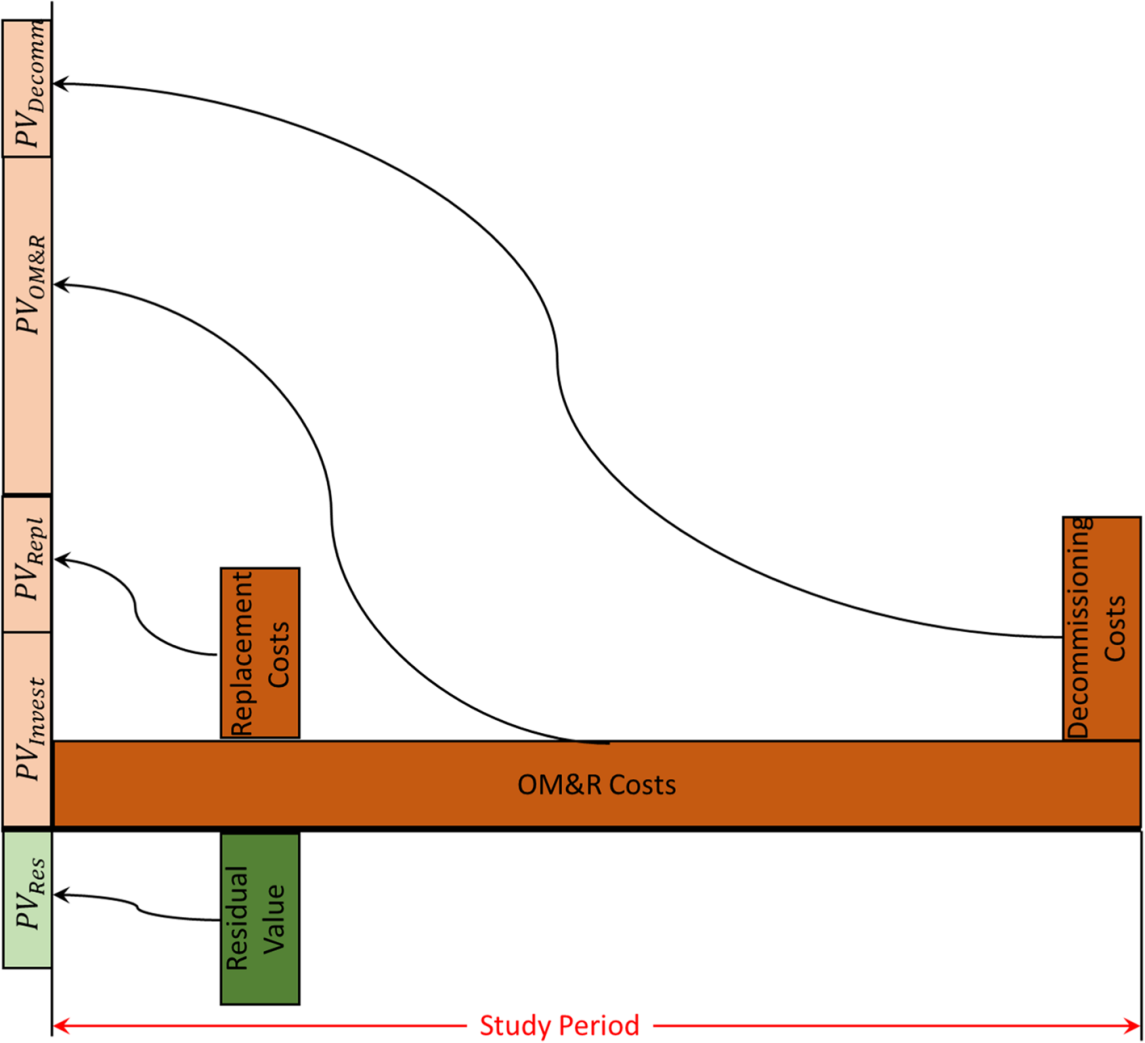
Estimation of lifecycle cost by adding present values of different costs

The planning-construction period spans from base date to service date. While base date refers to the instant to which all the costs are discounted in LCC analysis, service date refers to the instant when the building is put to use by the occupants. The service period spans from the service date till the end of study period, and is also called beneficial occupancy period. In simpler terms, while initial cost of construction and/or investment (Invest) is considered to be distributed over the planning-construction period, costs of operation, maintenance and repair (OM&R) are spread through the service period of the building. The costs towards energy consumption by the building is considered as an operating cost, and is therefore a part of OM&R costs. It should be noted that an LCC analysis considers costs and benefits involved with decommissioning (Decomm) at the end of the study period rather than at the end of design life. While demolition and disposal attract costs, scrap value of the building at the end of study period counts towards benefits or negative costs. If the planning-construction period is fairly small compared to the complete study period, an LCC analyst may consider zero duration for planning-construction period. In such case, the initial cost of construction and/or investment is lumped at the base date. The future OM&R costs are usually assumed to occur at the end of the corresponding year for simplicity [108, 109]. However, this convention may vary with agency, jurisdiction and analyst’s judgement. Each of the future costs (OM&R and Decomm) are then discounted to the base date, where the initial costs (Invest) are already estimated. LCC of the project is then obtained by summing the present values of all these components, evaluated at the base date. This is represented in Eq. (2) and Fig. 7. The general expression for estimating LCC at present value is summarized in Eq. (3) where *N* represents study period in years [108].

2 $$LCC={PV}_{Invest}+{PV}_{Repl}-{PV}_{Res}+{PV}_{OM\&R}+{PV}_{Decomm}$$

3 $$LCC=\sum_{t=0}^N\frac{FV}{{\left(1+d\right)}^t}$$

It is evident from Eq. (2) that there are more components of LCC than present values of Invest, OM&R and Decomm costs. In case of existing conventional structures being converted into passive buildings, replacing components of building envelope attract further costs. This cost is called replacement cost and is given by *PV*_*Repl*_ in terms of present value. The replaced component has some scrap value and is therefore a negative cost. This is usually termed as residual value and is given by *PV*_*Res*_ in Eq. (2). Equation (2) considers basic amenities like electricity and water as parts of operations, and therefore these costs are included in OM&R costs. It should be noted that some articles and manuals do not include electricity and water costs in OM&R costs, and rather add them separately.

Each of the future costs can be classified as either non-recurring or annually recurring costs. While non-recurring costs are discounted to base date using simple present value (*SPV*) factors, annual recurring costs are discounted to base date using uniform present value (*UPV*) factors. There can be some costs, such as repair costs, which are recurring but the periodicity is not annual. These costs are considered as non-recurring and are discounted to base date using *SPV* factors. The expressions for *SPV* and *UPV* factors are presented in Eqs. (4) and (5) respectively. These expressions consider non-recurring costs to be incurred at the end of year *t* and recurring costs to be incurred annually over a period for *n* years. Very often, recurring costs are not constant over time. For instance, the annual energy consumption of a building may stay same over years, but the power supplying grid is likely to increase its cost per unit leading to an increase in energy costs over the years. Such changes in recurring costs are accounted by use of rate of escalation denoted by *e*. Rate of escalation represents the projected increase in a recurring cost over the years. Since, for instance, electricity charges per unit escalate by a rate different from the general inflation rate in the region, escalation rate should not be confused with the general inflation rate. *UPV* factors are adequately modified to obtain modified uniform present value (UPV*) factors given by Eq. (6). Another possible change in energy cost over time can come from the composition of energy types. The whole world is steadily moving from non-renewable sources such as fossil fuels to renewable sources such as solar, for meeting its buildings energy needs [111]. If reliable past data is available, the change in energy sources over the study period should be projected for obtaining a better LCC estimate.

4 $$SPV=\frac{1}{{\left(1+d\right)}^t}$$

5 $$UPV=\sum_{t=1}^n\frac{1}{{\left(1+d\right)}^t}=\frac{{\left(1+d\right)}^n-1}{d{\left(1+d\right)}^t}$$

6 $${UPV}^{\ast }=\sum_{t=1}^n{\left(\frac{1+e}{1+d}\right)}^t=\left(\frac{1+d}{d-e}\right)\left[1-{\left(\frac{1+e}{1+d}\right)}^n\right]$$

*UPV* and UPV* factors inherently assume that the costs recur every year starting from the base date. However, when there is a planning-construction period, annually recurring costs usually start to incur from the service date. This issue is resolved by excluding those costs for the duration of planning-construction period, as shown in Eq. (7), where *t*_*PC*_ denotes the planning-construction phase in years.

7 $${UPV}^{\ast }=\sum_{t=1}^n{\left(\frac{1+e}{1+d}\right)}^t-\sum_{t=1}^{t_{PC}}{\left(\frac{1+e}{1+d}\right)}^t$$

It is worth mentioning that the process of discounting discussed so far, considers costs at current price levels. In simpler terms, a future cost incurred in 2030 is based on prices in 2030, which is then discounted to equivalent present value using discount rate. Such a discount rate is termed as nominal discount rate (*d*). However, there is another phenomenon called inflation which reduces the purchasing power of money over time. If inflation is excluded from the expected future costs at current price levels, future costs at constant price levels are obtained. In such a case, the discount rate needs to be adequately modified. Such a discount rate is called real discount rate (*D*). Similarly, nominal escalation rate (*e*) should also be replaced with real escalation rate (*E*). It should be noted that both approaches, when used with consistent assumptions about discount, escalation and inflation rates, will yield the same result for present value of any future expense. The two approaches are respectively termed as current dollar and constant dollar methods. The relationships between nominal and real discount and escalation rates are reported in Eqs. (8) and (9), where *I* denote the rate of inflation [108].

8 $$D=\frac{1+d}{1+I}-1$$

9 $$E=\frac{1+e}{1+I}-1$$

Though there are a number of resources available for estimating all these future costs in the United States [108–110], most other countries don’t have enough resources. In such cases, the analyst has to rely on projections from available data, quotations from vendors and own judgement. When estimating energy demand of the building, one needs to consider variations in average demand over seasons and peak demand as well. While converting future energy demand estimates to monetary values, one must consider variation in grid charges per unit with total monthly consumption as well as variation in grid charges with seasons. It should also be noted that resources based on data from one region should not be indiscriminately used in other parts of the world. This may render LCC estimation not very reliable, and final decisions must be made taking this in consideration.

An LCC analyst may come across numerous costs associated with ownership, operation, maintenance and decommissioning of a building. However, it may not be prudent to consider all project-related costs in an LCC analysis of multiple design alternatives. Based on experience and judgement, the analyst may decide to consider only those costs that are significant in quantitative measure and relevant to the decision at hand. Costs, which are expected to be approximately the same for all proposed design alternatives, should be omitted from LCC estimation in order to save costs on data collection and analysis. In case of an existing building with a proposal to incorporate passive elements, initial cost of structure (same for base case and all retrofit alternative cases) should not be considered as it would be a worthless exercise. However, installation of passive elements may need some existing elements to be removed. In such cases, in addition to costs of passive elements, costs of replacement and benefits of decommissioning existing elements should be considered in LCC estimation. In such retrofit projects, OM&R costs which have already been incurred should also be avoided while estimating LCC. These already incurred costs are called sunk costs. Costs which are likely to affect decisions insignificantly should also be avoided. For instance, an efficient plumbing network may require estimation of water costs but estimation of electricity costs is not required. Some project-related effects are difficult to quantify in monetary terms. For instance, installation of a passive element may improve the quality of life for occupants in addition to energy savings. The improvements in quality of life are difficult to quantify as it would require estimating increase in productivity, reduction in hospital visits and associated expenses, and a vast multitude of non-monetary benefits [112, 113]. Specialized literature with proposed quantitative metrics can be helpful in such cases [114–116]. In absence of any such clear metric, qualitative descriptions must be appended in an LCC report so that the stakeholders are able to arrive at an informed decision.

It must be evident to the readers that LCC estimation is helpful in assessing economic feasibility of multiple design and/or retrofit alternatives, and can assist the stakeholders select the alternative with the lowest LCC. However, a number of decisions may require supplemental measures. While this sub-section discusses some of the supplemental measures, the next sub-section outlines some of the common decisions to be made by the stakeholders and suitable quantitative measures.

### Supplemental measures for economic feasibility analysis

In addition to LCC, the most commonly used metrics to assess economic feasibility of design alternatives and/or projects are net savings (NS), savings-to-investment ratio (SIR), adjusted internal rate of return (AIRR), simple payback (SPB) and discounted payback (DPB). While judging cost-effectiveness of a passive building with respect to a conventional building, NS, SIR and AIRR yield same results as LCC. However, when assessing multiple project alternatives (design and/or retrofit) with an aim to select one from the lot, only NS is equivalent to LCC. In other words, SIR and/or AIRR may suggest a different alternative to be the worthiest for incorporation.

NS for a project design and/or retrofit alternative is obtained by subtracting the LCC of the alternative (A) from the LCC of the base case (BC), as given by Eq. (10). It is intuitive that NS can also be estimated from differences in individual cost categories as depicted in Eq. (11), or from savings and investments. Positive NS implies that the proposed alternative is cost-effective. While evaluating multiple alternatives, the alternative with lowest LCC will have maximum NS. It is worth noting that LCC and NS can be used interchangeably as they are entirely consistent. NS estimation has an added advantage that the analyst needs to consider only those cost components that are different for the base case and the alternative. However, unlike LCC, estimating NS requires identification of a base case among the alternatives. It should be understood that the base case and the alternative should have the same base date, study period and discount rate.

10 $$NS={LCC}_{BC}-{LCC}_A$$

11 $$NS={\left({PV}_{Invest}+{PV}_{Repl}-{PV}_{Res}+{PV}_{OM\&R}+{PV}_{Decomm}\right)}_{BC}-{\left({PV}_{Invest}+{PV}_{Repl}-{PV}_{Res}+{PV}_{OM\&R}+{PV}_{Decomm}\right)}_A$$

At times, investors in a building project are interested in predicting savings for every unit investment. For a given design and/or retrofit alternative, SIR is defined as the ratio of the present value of future savings to the present value of investment (all discounted to base date) over the base case. SIR can be considered as a benefit-to-cost evaluation metric. The base case and the considered alternative should have the same base date, study period and discount rate. A project alternative is economically justified if it has an SIR value exceeding unity. It is intuitive that SIR value of 1 and NS value of 0 are equivalent to the case where the alternative has the same LCC as the base case. Though the three definitions sound consistent, the alternative with lowest LCC and maximum NS may not have maximum value for SIR. This can be comprehended based on the principle of diminishing marginal utility. For instance, a double insulation layer on the building envelope with a lower SIR may be more cost effective compared to a single insulation layer on the building envelope. The general formula for obtaining SIR is given by Eq. (12).

12 $$SIR=\frac{{\left({PV}_{OM\&R}\right)}_{BC}-{\left({PV}_{OM\&R}\right)}_A}{{\left({PV}_{Invest}+{PV}_{Repl}-{PV}_{Res}+{PV}_{Decomm}\right)}_{BC}-{\left({PV}_{Invest}+{PV}_{Repl}-{PV}_{Res}+{PV}_{Decomm}\right)}_A}$$

AIRR is also a relative measure of economic feasibility which implies that the base case and the alternatives should have consistent base date, study period and discount rate. AIRR measures expected annual percentage yield from an investment over the study period. AIRR is compared with the discount rate to arrive at meaningful inferences. Since discount rate usually equals the minimum rate of return acceptable to the investor, AIRR exceeding discount rate implies that the alternative is economic. If AIRR is lower than discount rate, the investor would be happy investing the capital somewhere else and the concerned project alternative is not economically lucrative. AIRR calculation requires calculation of SIR, and is presented in Eq. (13).

13 $$AIRR=\left(1+r\right){(SIR)}^{\frac{1}{N}}-1$$

SPB and DPB quantify the time required to recover initial investment, and are expressed in number of years from the service date. In simpler terms, it is the time from the service date to a date when cumulative savings just offset the incremental investment costs. Since DPB considers discounting future cash flows to the present value, it is preferred over SPB. Since SPB and DPB are measured from the service date, they are compared against service period and not study period. For a project alternative to be economical compared to the base case, DPB needs to be lesser than the service period considered. This would make payback criterion of economic feasibility to be consistent with the LCC criterion. However, most investors would prefer DPB to be much smaller than the service period.

### Decisions related to economic feasibility

Till now in this section on understanding economic feasibility of passive buildings, we have discussed estimation of LCC followed by a number of supplemental measures. This sub-section outlines some of the decisions related to capital investment that are frequently encountered in case of passive buildings, along with the suggested metric.

#### Decision 1: accept or reject a design and/or retrofit alternative

Decisions such as whether to use cavity walls over regular masonry walls, to replace conventional single pane windows in an existing building with double pane windows etc., can be made based on estimation of LCC, NS, SIR and/or AIRR. Lower LCC for the alternative (compared against base case), positive NS, SIR greater than unity and AIRR exceeding discount rate typically imply that the project alternative is economically admissible and worth incorporating.

#### Decision 2: select an optimal efficiency level for a building envelope

A project may involve decisions such as whether to use a conventional masonry wall or a cavity wall with possible variation in cavity thickness. In this example, LCC and NS are suitable measures to choose what cavity thickness would be the most cost-effective, if cavity walls are cost-effective over conventional walls. Such decisions should not be made based on SIR and AIRR. It is because incorporation of passive elements follow principle of diminishing marginal utility and therefore these measures will tend to suggest solution with least cavity thickness.

#### Decision 3: select an optimal passive element from competing alternatives

This typically refers to a situation when the stakeholders have multiple design and/or retrofit strategies to choose from. For instance, an analyst may come across a decision to opt for a certain glazing for windows from a number of available alternatives. Such decisions are in a sense similar to decisions about selecting an optimal efficiency level. Therefore, LCC and NS are suitable measures for arriving at such decisions. SIR and AIRR should not be used to take such decisions.

#### Decision 4: select an optimal combination of interdependent active and passive elements

There can be a need to select an optimal combination of interdependent active and passive elements, for economic feasibility. For instance, since thermal insulation and available lighting affect energy demand for a building, thermal insulation of the building envelope, daylighting through the envelope and efficiency of the installed HVAC system are interdependent. LCC is the most suited metric in such situations. NS is equally good; however, one of the combinations needs to be identified as the base case. In most cases, highest SIR and/or AIRR or shortest DPB does not indicate the correct combination and hence should be avoided.

#### Decision 5: rank independent projects in a larger project lot so as to allocate funds from a limited budget

The previous decisions pertained to selection of the most cost-effective choice among a number of mutually exclusive design and/or retrofit alternatives. However, there can be situations where independent projects in a project lot are to be ranked so as to allocate funds from a limited budget. For instance, if five residential buildings worth amount X in total are to be built but the client has only 0.7X of available capital, the analyst may be asked to rank the five buildings for their worthiness towards allocation of funds. SIR and AIRR are the most suited metrics for this purpose [117, 118]. Projects with larger SIR and/or AIRR are likely to receive funds from the limited capital budget.

From the above discussion, it is evident that LCC and NS are the most versatile tools to assess economic feasibility of a passive building project. When a number of independent passive building projects are to be funded from a limited budget, the projects should be ranked using SIR and AIRR. The payback measures, SPB and DPB, are primarily used as screening tools to arrive at a limited number of project alternatives to be evaluated for economic feasibility. This is because of lack of consensus on desired payback time as most investors want payback time to be much lesser than the service period. Therefore, the use of SPB or DPB should precede the use of LCC, NS, SIR and/or AIRR. Table 1 summarizes suitability of different metrics for different decisions discussed in this sub-section.

**Table 1:** Suitability of different measures for different decisions outlined in Decisions related to economic feasibility section

Decision	LCC	NS	SIR	AIRR	DPB / SPB
**1**	Yes (Minimum)	Yes (> 0)	Yes (> 1.0)	Yes (>Discount rate)	No
**2**	Yes (Minimum)	Yes (Maximum)	No	No	No
**3**	Yes (Minimum)	Yes (Maximum)	No	No	No
**4**	Yes (Minimum)	Yes (Maximum for combined NS)	No	No	No
**5**	No	No	Yes (Descending Order of SIR/AIRR until budget is exhausted)	Yes (Descending Order of SIR/AIRR until budget is exhausted)	No

## Climatic adaptability of passive buildings

In 2000 and 2010, more than 95% and 70% of all passive buildings were located in Germany and Austria respectively [119]. Though the concept seems to be spreading out, the spread has been mostly limited to Europe [119]. There can be diverse reasons for this poor acceptability of passive buildings outside Europe especially in Asia and Africa. Most of the construction in Asia and Africa tend to reduce the initial cost of construction. As passive buildings are usually expensive than conventional ones, they are not sought in most cases. The other reason may be the extreme climate conditions prevailing in Asia and Africa. While passive buildings in Europe aim to achieve heating of indoor environment without over-reliance on active measures, such buildings in Asia and Europe have to achieve heating in winters and cooling in summers. This contrast in desired features of a passive building is difficult and more expensive to implement, and thereby passive buildings are not gaining enough popularity in tropical and temperate climate zones. It is also worth noting that most of the passive buildings in Asia and Africa are public buildings, unlike residential buildings in Europe [119]. This suggests that governments in these regions are keen to achieve energy conservation and emission reduction in buildings and building construction industries.

There is requirement for passive buildings to meet diverse loads, i.e., heating and cooling loads in winters and summers, in large parts of the world. Further, passive buildings in different climatic zones have to cater to different heating, cooling and ventilation requirements. Despite differences in goals of passive buildings located in different climatic zones, the fundamental principle remains the same. In other words, the situation can be summarized as same physical equations with varying boundary conditions. Passive buildings in cold and hot climates have heating and cooling loads respectively. The common objective is to reduce the peak load drastically through incorporation of passive mechanisms in the building envelopes. These mechanisms include building insulation, passive solar gain, heat recovery and other measures. In cold weather, the peak heating load should be restricted to 10 W/m^2^. This implies that when heating load to ensure comfortable indoor living during winters reaches around 10 W/m^2^, the ventilation system should be able to heat the indoor spaces and not allow the heating load to soar up. Similarly, in hot weather, the peak cooling load should be adequately restricted beyond which ventilation system should be capable of cooling the indoor spaces. In places with very high relative humidity, indoor environment may require dehumidification for comfortable living. When dehumidification load reaches a limit, ventilation system should be capable to restore ambient humidity using dry air circulation.

Tropical climate is characterized by high temperature and high relative humidity. Cooling load in such places can become excessively high, which can be subdued only through proper ventilation. Therefore, the most useful passive design strategy is to maximize cross-ventilation and convective air flow. For industrial sheds and warehouses, clerestory windows located at higher levels can help hot air to escape. Wind-driven roof vents are also commonly used in industrial buildings to facilitate escape of hot air. These vents spin faster when outside temperature is higher, and do not need electricity for running. Further, lightweight materials with low thermal conductivity (such as fly ash blocks) should be used for walls and roofs. Cavity walls and ventilated roofs are suitable solutions to minimize solar gains. Solid walls made of heavy weight materials like clay bricks and concrete should be well-shaded as far as possible. Openings should have sunscreens to prevent direct solar gains. Chajjas (overhangs), pergolas and jaalis (perforated screens) are commonly used to screen away direct sunlight. Glazings in openings should have low solar heat gain coefficient (SHGC) and high visual light transmission (VLT), to ensure low heating and high daylight at the same time. Passive building designers usually aim for high ratio of VLT to SHGC. Ecological features like vegetation and lakes around the building are also go-to passive strategies as they act as heat and carbon sinks. Supply air ducts to cool and dehumidify the indoor environment are usually used. Air flow in supply air ducts consumes much less energy than using active measures like HVAC systems. Traditional construction in some regions include air channels and buried ducts, and should also be explored. There have been numerous studies for passive building design in tropical and sub-tropical climate [16, 18, 120–131] and arid climate [132–134] zones.

Temperate climate is characterised by hot summers and cold winters. This poses contradictory requirements for summers and winters. Passive buildings in temperate climate have to cater to cooling load in summers and heating load in winters. This is achieved by positioning windows on southern face in northern hemisphere and northern face in southern hemisphere. Since the relative humidity in temperate climate is usually low implying low dehumidifying load, ventilation is not the primary requirement. Buildings in such climate therefore need good insulation against extreme outdoor temperatures. Contrary to tropical climate, passive buildings in temperate climate should use heavy weight materials with high thermal mass such as concrete and clay bricks for their walls and roofs. Cavity walls and ventilated roofs are however equally suitable in temperate climate. Proper orientation of buildings, careful design of its eaves and sunscreens and appropriate positioning of windows are important. The objective should be to allow winter sun to heat the indoor spaces while preventing the summer sun from entering inside the building. A number of researchers have attempted to obtain working design of passive buildings in temperate climatic regions [135–140].

In cold climates, passive buildings need to heat the indoor environment throughout the year without excessively relying on active heating measures. This is achieved by properly insulating the building envelope and maximizing solar gain. Solar gain is maximized by placing large glass openings on southern and northern faces in northern and southern hemispheres respectively. Since the outdoor temperature can go extremely low, insulation of building envelope should include thermal breaks and avoid thermal bridges. Thermal breaks are usually made of insulating material as shown in Fig. 5. Different studies to improve efficiency of passive buildings in cold climates have been conducted [141–144]. Some regions can have composite climates where there is large variation across the different months of a year. Designing passive buildings in such regions is challenging and some recent studies have been conducted to address this [145–149].

In case of large projects such as mass housing and public structures where large capital is required, design of passive buildings needs to consider local climatic variations as precisely as possible. This enables architects and designers in achieving a passive building which provides comfort to its occupants while being energy efficient. Such design problems are usually called as climatic adaptation problems, and can be resolved through design parameter optimization where the designer tends to optimize design parameters related to building envelope such as heat transfer, heat capacity, air tightness and ventilation [12, 150–163].

Since passive buildings involve initial investment, another concern is their ability to adapt to changing climate. Most of the regions around the world are experiencing climate change and global warming. A number of researchers have been recently assessing adaptability of passive buildings to climate change in different parts of the world [164–172].

The aforementioned literature in this section helps us comprehend and consider parameters that are suitable to the regional climate and possibly accommodate to the climate change. However, the stakeholders including owners and construction companies will be confident about adopting passive buildings if the respective government prescribes standards and guidelines in this context. The upcoming section reports some of the prominent standards related to passive building construction from around the world.

## Standards on passive buildings

Most of the codes and standards related to passive building construction can be classified as either prescriptive, performance-based or outcome-based [173, 174]. A prescriptive standard enlists materials and equipment for walls and roof with certain properties such as *R* and *U* values in order to make the building energy efficient. However, orientation of the building and its openings to incorporate desired solar gain and ventilation is not considered. Performance-based standards establish a baseline system and compares the designed system against the baseline system. These standards therefore provide opportunities for trade-offs across multiple systems to arrive at the most cost-efficient system while achieving desired passive building performance. The outcome-based standards establish a target energy consumption level and suggest measures to achieve the established level while providing designers enough flexibility.

The most prominent specifications related to passive buildings come from Passive House Institute (Passivhaus Institut in Deutsche, PHI) [175]. Passive House Institute has several sister organisations around the world, such as Passiv Haus Institut (PHI), Passive House Institute (PHIUS), Canadian Passive House Institute, Australian Passive House Association, Passive House Institute Japan (PHIJP), Hellenic Passive House Institute and Passive House Institute Italia, who are committed towards passive house general approach and building standards in their respective regions. Apart from certifying design professionals, builders and products, these organisations train people and conduct fundamental research in modelling of passive buildings. They also publish standards for constructing passive houses.

**Fig. 8:**
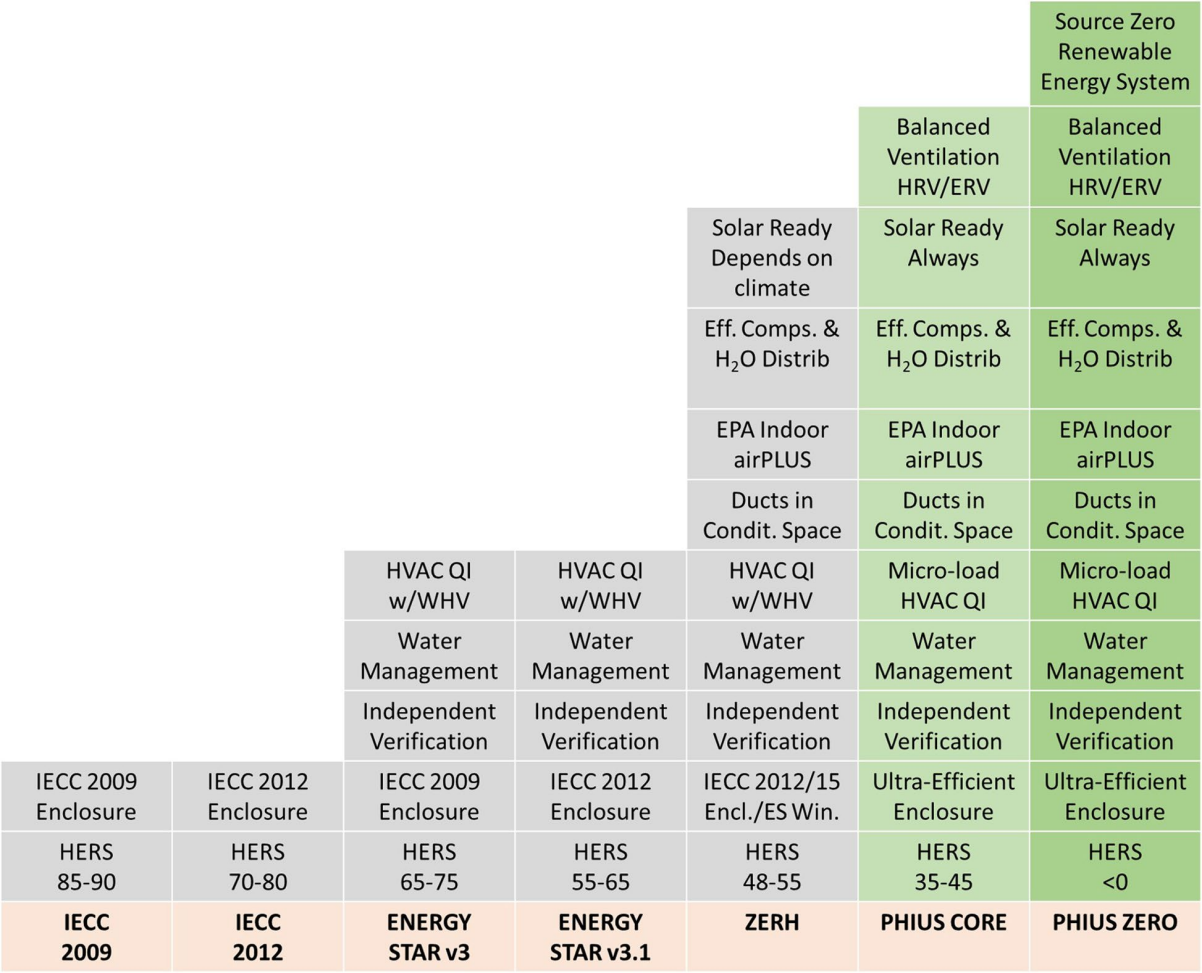
Advancement of PHIUS CORE and PHIUS ZERO standards over previous building codes

PHI standards have been around since mid-1970s when there was a move to construct some energy efficient buildings during energy crisis in the United States. Over the coming decade, first energy reduction targets were set for such buildings in the United States and Canada, as 15% of the typical heating loads. The standard got adapted in many European countries over time. By 1996, the Passive Haus Standard in Germany became quite rigorous. During this time, popularity of passive buildings was localised in Europe. Over last two decades, passive buildings have been slowly gaining foothold in other regions of the world especially in North America. Later on, PHIUS devised two major passive building standards, called PHIUS CORE and PHIUS ZERO [176, 177]. These were major advancements over previous building codes from different organisations including International Energy Conservation Code (IECC) 2009 and 2012, Energy Star v3 and v3.1, and ZERH, as shown in Fig. 8.

PHIUS CORE and PHIUS ZERO has two basic strategic components, viz. energy model performance and on-site quality assurance via testing/inspection. The main certification requirements include various targets to be reached. While air tightness needs to be restricted to 0.060 cubic feet per minute for every square feet of envelope area to change building pressure by 50 Pa, space conditioning targets (including heating and cooling demands) are based on cost optimization, climate, occupant density and envelope to floor area ratio. PHIUS also provides a calculator to obtain space conditioning targets depending on aforementioned factors. The net energy target should be checked against energy demand at the source rather than energy consumed at the site, to obtain a better estimate for carbon emissions. Site energy demand can be estimated from site energy demand using source energy factor, which in turn depends on energy source. While PHIUS CORE sets certain limits on source energy demand for buildings, PHIUS ZERO prescribes net zero source energy demand over a year. In order to achieve these targets, buildings may need to employ both active and passive strategies. Additional quality requirements include regulation of combustion-venting systems and use of building materials with low emissions. These standards also include climate-specific guidelines which is important as discussed in the previous section. The most commonly implemented guidelines are prescriptive, which comprise of criteria listed in Table 2.

**Table 2:** Prescriptive path to PHIUS certified passive buildings

Criteria	Description
Compactness	To prevent non-compact designs, by restricting enclosure area based on geometry of the buildings
Solar protection	To achieve optimal solar heat gain through appropriate fenestration orientations and overhangs To limit high peaks in cooling load in summers, and to limit net heat loss in winters Different limits set for different climate zones, using regression
Thermal enclosure	To set maximum U-value for fenestration and minimum R-value for walls and roofs, using climate-dependent regression formulae
Mechanical ventilation	To set climate-specific limits on ventilation to obtain continuous fresh air supply while achieving required thermal enclosure
Mechanical systems	To use energy efficient air source and ground source heat pumps, depending on climate zone of the site
Lighting, appliances and water heating	To use energy efficient electrical appliances, usually in compliance with ENERGY STAR or ENERGY STAR Most Efficient 2020 appliance ratings

On a general note, the upgradation from PHIUS CORE to PHIUS ZERO can be understood as the following remark. In order to get a building certified with PHIUS CORE, one needs to ensure quality and durability of structure, and to employ passive and active energy conservation strategies. Further, to get certification from PHIUS ZERO, the stakeholders have to explore on-site renewable energy sources and even off-site renewable energy sources in some cases. The hierarchy is to explore measures in the following order: passive energy conservation, active energy conservation, on-site energy generation from renewable sources and off-site energy generation from renewable sources.

Till the end of 2021, over 950 projects with a total of over 13,000 housing units have been submitted for obtaining certification from PHIUS. There has been an exponential growth in buildings applying for certification every year. The PHIUS certification guidebook contains guidelines for certifications depending on project type, viz., single-unit and multi-unit residential buildings, community buildings, commercial buildings and multi-use buildings.

Standards from PHI organisations in other regions are similar to PHIUS CORE and PHIUS ZERO. They aim to lower energy consumption of buildings by means of five key principles, viz. compact building form, super-insulated building envelope with continuous control layers, balanced ventilation with minimal mechanical systems, high-performance windows, and air-tightness. These principles become most critical at the boundaries of various elements such as connections between walls and windows. This is achieved by introducing thermal breaks and avoiding thermal bridges. The international building criteria from PHI typically include limits on heating demand, cooling demand, source energy demand and airtightness. While annual heating demand is restricted to 15 kWh/m^2^, annual cooling demand is limited to 70 kWh/m^2^ depending on climatic zone. The primary source energy demand for a year should not exceed to 120 kWh/m^2^. Airtightness of the building should be restricted to 0.6 air changes per hour at pressure of 50 Pascals. These standards also evaluate climate-specific energy demand which is important as discussed in the previous section [178].

The common challenges for implementation of passive building specifications are differences in climate, culture and construction. In case of emerging economies like India, there are other challenges such as lack of required awareness, knowledge and training among individuals, and unavailability and/or unaffordability of components. Construction and monitoring of pilot projects are likely to be helpful in designing guidelines for these regions. Further, passive building regulations should be accompanied with adequate incentives. Traditional construction methods should also be scientifically tested to achieve energy efficiency. This will enable the local artisans and masons, and also make the building affordable and energy efficient. Collaboration of local and international experts with construction industry practitioners is also required to achieve wide popularity of passive buildings.

Apart from passive buildings, there has been a global impetus towards net zero energy buildings. The energy produced from renewable sources by these buildings over a year is equal to their annual energy demand. California Public Utilities Commission, the leading body for Net Zero Energy Building (NZEB) policies in California, aims to have 100% of new and 50% of existing commercial construction in California to be net zero energy. At the same time, it also has a target of all new federal buildings to be net zero energy. Building Energy Efficiency Program, Appliance Efficiency Program, Non-Residential Building Energy Use Disclosure Program, California Green Building Standards and Zero Net Energy Pilot Programs are major initiatives in this direction. Across the nation, U.S. Department of Energy has been actively organising competitions, award functions and other events aimed at spreading awareness towards energy efficient buildings.

In Europe, European Union issues Directive on Energy Performance of Buildings (EPBD) periodically, which sets NZEB goals and binds member nations to achieve them [179]. There are other directives such as Renewable Energy Directive, Energy Service Directive Energy Efficiency Directive, Eco-design Directive and Energy Labelling Directive. However, member nations are free to set their interim targets. For instance, Germany has set target reduction of 80% in primary energy requirement and has aimed to achieve climate-neutral building stock by 2050.

In Japan, all buildings, including new and existing ones, are set to be net zero energy by 2050. Voluntary building standards for both residential and commercial buildings are regularly strengthened in this context. Comprehensive Assessment System for Building Environmental Efficiency (CASBEE) is a green building rating system which evaluates environmental performance of buildings, and has been made mandatory by over 24 local governments in Japan [180]. The country also has system of loans, grants and tax incentives under Low Carbon Cities Promotion Act, Eco-point Housing Program, Zero-Energy Housing Grant Program and other subsidy programs, to provide impetus to construction of energy efficient buildings.

In particular to India, “Net Zero Energy Buildings” is an alliance of multiple organizations aiming to accelerate market development of NZEBs in India, with a target of making them affordable by 2030. It aims to achieve this through innovation and research in design and construction practices related to NZEBs along with skill development of the workforce. It derives inspiration from aforementioned policies and case studies related to NZEBs adopted in different regions of the world. A marvel of NZEB implementation in India is the Nalanda University [181].

There are a number of rating systems which rate buildings as per their energy efficiency. The most prominent among them are Home Quality Mark Standard from The U.K. Building Research Establishment Environmental Assessment Method (BREEAM) [182], Leadership in Energy and Environmental Design (LEED) Green Building Rating System from U.S. Green Building Council (USGBC) [183], EnerGuide Rating System (ERS) from Sustainable Buildings Canada (SBC) [184], Home Energy Rating System (HERS) from Residential Energy Services Network (RESNET) [185], and Energy Conservation Building Code (ECBC) from Bureau of Energy Efficiency (BEE) in India [186].

**Fig. 9:**
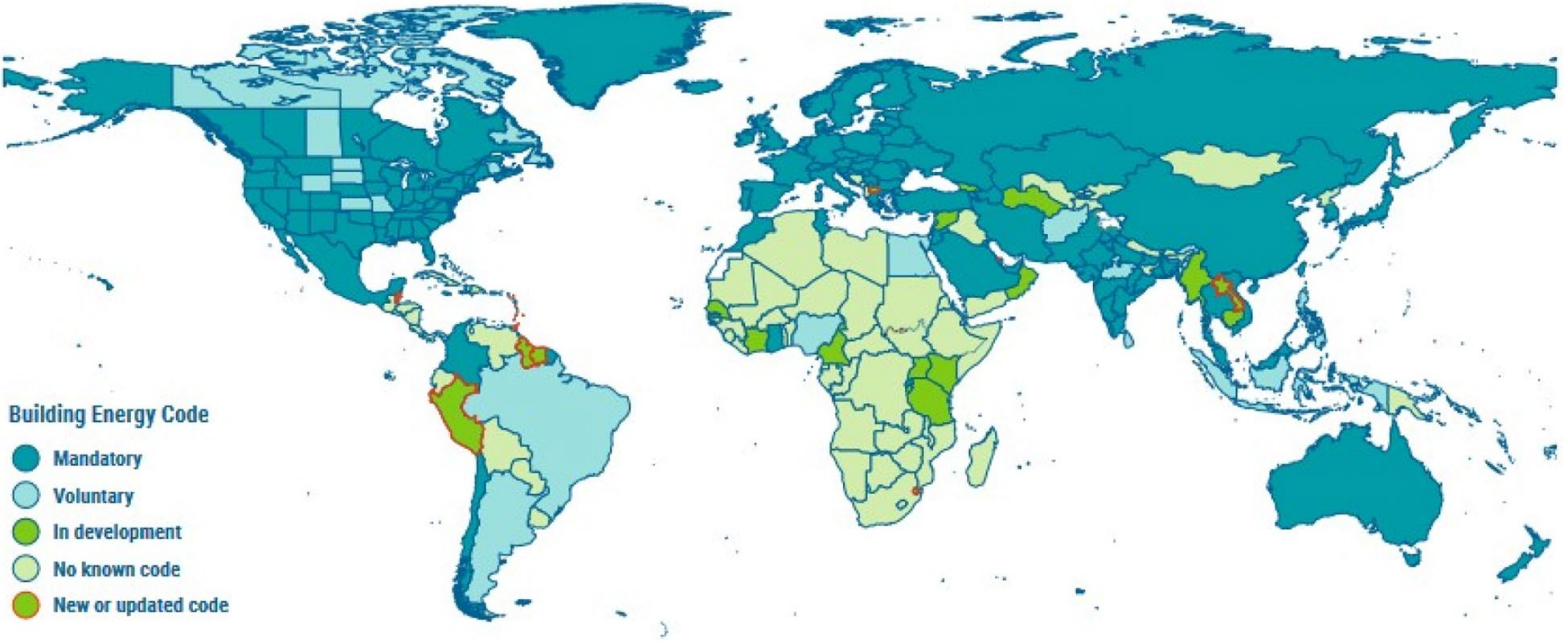
Status of building energy codes across different nations and states [4]

A number of countries are gradually coming up with their own standards on passive house construction based on their climates [187]. This is evident from Fig. 9 [4]. However, most parts of the world are yet to initiate the process. Therefore, passive building designers have to rely on specialist literature in order to comprehend economic feasibility and climatic adaptability. In this context, a large number of relevant past literature are cited in the previous two sections. Since passive buildings are an evolving domain with huge scope of innovations, the upcoming section contains recent research trends in its various sub-domains. This will help novice researchers in finding research gaps and initiating their research, and practitioners in getting familiar with recent advancements.

## Contemporary research

It is evident from previous sections that passive buildings are likely to be answers to menaces of energy crisis and emissions originating from buildings and building construction industries. Standards from different Passive House Institutes have set up rigorous requirements in this context, such as reducing heating and cooling demands by 90% and 75% respectively. Since this goal is not possible to achieve using active measures like efficient lighting, HVAC and plumbing systems, passive measures such as changes to building envelope become essential. Development of these modifications in building envelopes requires innovation, simulation and testing [188]. The first step is to conceive a plausible concept based on knowledge from multiple disciplines such as thermodynamics, planetary science and climate science. The conceived concept is then numerically simulated to understand possible efficiency of the concerned modifications. If the concept is found worth, experiments are conducted to test the actual efficiency of planned passive strategies. Since experiments can be cumbersome and expensive, the simulation step should be used to reject unviable strategies.

It should be understood that both simulation and testing should preferably be performed on full building model rather than just on components. Different combinations of components can be used to create different building models, so as to arrive at the most economically feasible and climatically suitable solution. These are called Building Envelope Optimization (BEO) problems, and are usually multi-objective in nature. While some studies pertain to hypothetical structures to assess efficacies of devised passive strategies [189–204], others analyse performance of constructed passive buildings [205–210]. Despite these advances, there are a lot of research gaps in the domain of BEO [211].

Many researchers have also been actively working on simulation programs and tools used to solve multi-objective building envelope optimization problems [212–216]. Some of the commonly used simulation tools are BLAST, BSimDeST, DOE-2.1E, Ener-Win, EnergyPlus, eQUEST, SUNREL, TRNSYS, Lightscape and DesignBuilder.

Despite advances in simulation programs to estimate efficacy of passive building design, testing the concept passive building is equally important. This requires a multi-disciplinary team. Efficacy measurement in practice requires installation of a sensor network system capable of quantifying comfort of occupants. A lot of recent research has focussed on building energy management system (BEMS) using sensor network and Internet of Things (IoT) [217–224]. Using advanced sensor network, it is possible to monitor and quantify different parameters such as indoor temperature, speed of wind outside the building and real-time energy consumption. Using IoT, adaptable system architecture can be established which can make use of sensor data to improve the performance of the passive building [225]. Designing an efficient sensor network with IoT for a given building still depends on expertise and experience of the multi-disciplinary team.

## Summary

This article presents a review of passive buildings from different perspectives, viz. technical design, economic feasibility, climatic adaptability, present state of practice and novel research opportunities. To summarize, passive buildings exhibit low energy consumption through compact building form, super-insulated building envelope with continuous control layers, balanced ventilation with minimal mechanical systems, high-performance windows, and air-tightness. These characteristics are achieved using diverse materials and technologies in the building envelope. The building envelope largely comprises of walls and roofs, and openings such as doors and windows. Owing to their low energy demand, these buildings are greener than conventional buildings.

Moreover, low energy consumption has a potential to retrieve the initial investment. This economic feasibility can be assessed by estimating the lifecycle cost in terms of present value. While the initial cost stays the same, the future costs such as those involved in operations, maintenance and disposal are brought to their respective present value through discounting. The lifecycle cost is usually considered over a specified study period. A number of supplemental measures of economic feasibility is also discussed in the article.

Since passive buildings aim at occupants’ comfort while being energy efficient, local climate has huge impact on requirements of such buildings. As a result, building envelope parameters need to be optimized in order to achieve desired results. Climate change adds another challenge as the climatic conditions are likely to change over the study period.

A number of prominent passive building standards and energy rating systems are also outlined in the article. These standards vary from prescriptive to performance-based and further to outcome-based. While a number of countries are making strides towards developing their own standards in order to make passive buildings acceptable and popular, a large number of countries are yet to initiate their moves. However, there is a common consensus that passive buildings are a solution to menaces of energy crisis and emissions from buildings and building construction industries.

It is also well understood that design of passive buildings is a multi-objective multi-constraint optimization problem. While reduced lifecycle costs, comfort to occupants and enhanced functionality are significant objectives, economic feasibility, climatic adaptability and ease of construction are major constraints. Considering the multi-disciplinary nature of these objectives and constraints, researchers from diverse disciplines have been actively working towards simplifying design of passive buildings. This article also contains some of the most active research trends in passive buildings, and cites recent and relevant articles. This is likely to be useful to novice researchers and practitioners. Greater the research in passive buildings and allied domains including wireless sensor networks and Internet of Things (IoT).

## Data Availability

Data sharing is not applicable to this article as no datasets were generated or analysed during the current study.
